# Autoantigenic nuclear proteins of a clinically atypical renal vasculitis

**DOI:** 10.1186/1740-2557-5-3

**Published:** 2008-07-14

**Authors:** Julio Avila, Elisa Acosta, María-del-Valle Machargo, María-Francisca Arteaga, Eduardo Gallego, Haridian Cañete, José-Javier García-Pérez, Pablo Martín-Vasallo

**Affiliations:** 1 Departamento de Bioquímica y Biología Molecular, Laboratorio de Biología del Desarrollo, Universidad de La Laguna, 38206, La Laguna, Tenerife, Spain; 2 Servicio de Nefrología, Hospital Universitario Nuestra Señora de Candelaria, Santa Cruz de Tenerife, 38010, Tenerife, Spain

## Abstract

**Background:**

Systemic vasculitides constitute a heterogeneous group of diseases of autoimmunological origin characterized by inflammation of blood vessels and antibodies that react against autoantigens in a process that ultimately affects blood vessel walls. An important number of these patients present kidney disease. An endeavour of this area of research is the identification of autoantigens involved in these diseases. Accordingly, we used serum from a patient suffering from a microscopic polyangiitis, P-ANCA positive, manifesting a clinically atypical renal necrotizing glomerulonephritis and interstitial nephropathy for the identification of autoantigens; we also determined the prevalence of corresponding autoantibodies in other vasculitides, diabetic microangiopathy and in general population.

**Methods:**

The patient's serum was used as a probe for the immunoscreening method SEREX to screen a human brain cDNA expression library.

**Results:**

Four positive clones were isolated and sequenced. Clones Jos002 code for protein HDAC5, Jos014 for TFC4, Jos107 for RTF1, and Jos313 for POLDIP3 polymerase. The four proteins are of nuclear localization. None of them had been reported as autoantigen. Recombinant proteins were synthesised and checked as antigens by western blot with different sera from controls and patients affected with other vasculitides and diabetic microangiopathy as well. Only the serum from the patient origin of this study recognized all recombinant proteins.

**Conclusion:**

We identify four nuclear proteins, HDAC5, TFC4, RTF1 and POLDIP3 polymerase as new autoantigens that could be used as markers in the diagnosis of subfamilies in immune diseases, although we cannot determine the role of these proteins in the aetiopathogenic process.

## Background

Systemic vasculitides constitute a heterogeneous group of diseases characterized by inflammation of blood vessels. Most of them are of autoimmune origin in which circulating antibodies are generated against autoantigenic proteins of the patient [1]. Classification of the disease is mandatory, by the size of the affected vessel, for the assessment of prognosis and the institution of treatment. The clinical presentation of vasculitis depends on the vessels involved. An important group includes alteration of kidney micro vessels in form of glomerulonephritis [2]. Spite of the broad amount of cases and the attributable pathogenesis of the disease to self-antigens, very few autoantigens related to this group of diseases have been found and described [3,4].

In the present work we have studied the immunoreactivity of the serum of a patient under no treatment, suffering from a vasculitis characterized by segmental and focal necrotizing glomerulonephritis with no immune deposits. We have identified four nuclear proteins that previously were unknown as autoantigens and that could be involved in the biological development of this vasculitis in which the presence of auto-antibodies is a dominant feature. We have used an immuno-screening method so called SEREX (serological identification of antigens by recombinant expression cloning). SEREX was initially employed to characterize tumour-expressed antigens eliciting an immune response in patients. This method, basically, consists of screening cDNA expression libraries with the selected serum to obtain purified clones to subsequent identification [5].

## Methods

### Patient data and Sera selection

The serum, taken at the time of biopsy, used as probe for the screening was obtained from a male patient 18 years old suffering from a microvasculitis diagnosed as MPA-microscopic polyangiitis with renal affection typed as focal-segmental necrotizing glomerulonephritis. P-ANCA positive and reactivity against MPO, C-ANCA was negative, serum was not reactive against PR3, ANA negative, and C3- and C-4 complement fractions were always within normal levels. Creatinine clearance at that stage was 37 ml/min. Diagnostic was qualified as "clinically atypical renal vasculitis". C-ANCA and MPO reactivity were negative after treatment with cyclophosphamide plus corticosteroids. Spite of the rapidly progressive glomerulonephritis, 6 years later, the patient still keeps a renal function of about 40 ml/min. For this study, eighteen healthy people (laboratory personnel), ten patients suffering from vasculitides and five diabetic microangiopathy with renal glomerular affection were selected as controls. Control vasculitides were CSS, WG, PAN, SLE and other MPA. Neither patients nor controls were undergoing any pharmacological treatment at the time blood samples were taken. In our study, sera from patients with vasculitis were used as positive controls for ANCAs, and samples from healthy people were assayed as negative ones. All of them were informed of the study and study was approved by the Ethical Committee of the Hospital Universitario Nuestra Señora de Candelaria, Tenerife, Spain, in compliance with the Helsinki Declaration. Psychological evaluation described the personality of the patient as 'slightly immature for the age'.

Methods used are described in Sambrook et al, 1989 [6], briefly as follows.

### Immunoscreening

A human brain cDNA expression library (Clontech, CA, USA) was used to perform the screening. The serum used as probe was diluted to a final concentration of 1:2000 in TBS, 0.05% Tween 20^©^, 3% bovine serum albumin and 0.01% sodium azide.

### Sequencing and alignments

DNA sequencing was performed by Sistemas Genómicos (Valencia, Spain). Clones for sequencing were prepared with the Qiaprep kit (Qiagen, Germany) following the instructions of the manufacturer. Sequence alignment with those in the GenBank, EMBL, DDBJ and PDB databases were performed using the BLASTN and BLASTX algorithms [8].

### Recombinant protein production

Selected cDNA clones (Jos002, 014, 107 and 313) were cloned in expression vectors pRSET A/B (Novagen, Germany). Expression of the recombinant proteins was allowed to proceed for 3–4 hours post-induction with 1 mM final concentration of IPTG in the *E. coli *BL21 (DE3) pLys-S bacterial culture. The bacterial cells were harvested, sonicated, and the fusion proteins were purified using kit Mg MagneHis™ Protein Purification System (Cat. #V8500, Promega).

### Western-blot

The recombinant proteins were electrophoresed on a denaturing 15% polyacrylamide gel and transferred to Immobilon™-P membranes (Millipore, MA, USA) by electroblotting. After blocking the filters with PBS, 0.1% Tween 20^©^, 5% dry milk, were incubated with 1:5000 sera from patients and controls in PBS, 0.1% Tween 20^©^, 0.5% dry milk for one hour. After washing, the blot was incubated with secondary goat anti-human IgG conjugated to horseradish peroxidase (Boehringer Mannheim, IN, USA) diluted 1:10 000 in PBS, 0.1% Tween 20^©^, 0.5% dry milk for 30 minutes. Detection was performed using ECL plus (Amersham Pharmacia Biotech, England) reagents according to the manufacturer's instructions. A different western blot membrane containing the four recombinant proteins was used for each patient or control.

### Expression patterns

Expression of the four autoantigens in different tissues was analyzed by Virtual Northern blot in the Genomics Institute of the Novartis Research Foundation web [7].

## Results and discussion

### Cloning, denomination and expression of auto antigenic proteins

We performed a SEREX immunological screening using as probe the serum of a patient suffering from focal-segmental glomerulonephritis. First name of this patient is Josué, based on this fact we decide to name clones 'pJos' and correspondent proteins 'Jos' followed by the number of the clone. Trough this method, we obtained four plaque pure clones which cDNAs were subcloned in pBluescript KS (plasmids pJos002, 014, 107 and 313) and sequenced. Aligning of sequences with those in GenBank, EMBL, DDBJ and PDB databases was done using the BLASTX and BLASTN algorithms [8].

#### Jos002

This sequence is 100% homologous to the human *HDAC5 *(*histone deacetylase 5*) and ortologue to *Hda1p *of *Saccharomyces cerevisiae*. Histone acetilation and deacetilation is part of a fundamental mechanism of gene expression control. HDAC5 belongs to HDACs IIa group which plays an important role by controling interactions among DNA and transcription factors and have implicated as global regulators of gene expression during cell differentiation and development [9]. McKinsey *et al*, suggest a role as signal-responsive regulators of antigen presentation to class II histone deacetylases when associated with ankyrin-repeat proteins [10].

#### Jos014

This sequence is homologous 100% to human *FLJ16014 fis *(*full insert sequence*) cDNA which is highly homologous to *TFC4 *(*Transcription factor 4, isoform b*). Presents a helix-turn-helix motif that binds to E-box (*Ephrussi*), 'CANNTG'. This sequence is among the first domains described for immunoglobulin stimulators. TCF4 is mostly expressed in immature lymphocytes B and possesses several mRNA species, although isoform b is the only one complete. Very recently has been reported that Tcf4 protein is involved in differentiation of neuronal progenitors and that haploinsufficiency of TCF4 causes the Pitt-Hopkins mental retardation syndrome [11]. This fact could be related to the mental retardation of the patient subject in this study, although in this patient Pitt-Hopkins syndrome was discarded.

#### Jos107

is homologous 100% to human RTF1/Rtf1 cDNA, Paf1/RNA polymerase II complex component [12], also called KIAA0252 and GTL7. RTF1 codes for a protein involved in regulation of transcription, chromatin structure and histone metilation in *Drosophila *through several independent functional domains [13-15]. Presents a PSSMs domain (*Plus-3*) 90 residues long, frequently associated to the *pfam02213 domain*, possess three residues with positive charge which is the origin of its denomination and that could be responsible for DNA binding.

#### pJos313

This sequence is homologous to human POLDIP3 polymerase (DNA-directed), delta interacting protein 3, also called SKAR, PDIP46 and KIAA1649. POLDIP3 binds to DNA and RNA during replication process, is a specific target of S6 kinase 1, interacts with p50 subunit of DNA polymerase delta, possess duplex DNA-unwinding and ATPase activities and regulates cell growth [16-18].

The four autoantigenic proteins reported are of nuclear localization.

Sizes of the above mentioned clones, genes that encode them, chromosome in which they are and data of the proteins are shown in Table 1.

**Table 1:** Some characteristics of genes and encoded proteins corresponding to isolated clones.

**CLON**	**SIZE**	**GENE**	**CHROMOSOME**	**ENCODED PROTEIN**	**N° OF AA**	**MW (kDa)**
Jos002	1000 pb	HDAC5	17q21	**HDAC5** *Human *Histone deacetylase 5	1122	122
Jos014	719 pb	TFC4	18q21.1	**TFC4** Transcription factor 4 isoform b	667	71.3
Jos107	800 pb	RTF1	15q.15.1	**RTF1** Paf/RNA polymerase II complex component	429	49.1
Jos313	1000 pb	POLDIP3	22q13.2	**POLDIP3** DNA polymerase delta interacting protein 3	421	46.1

**Figure 1:**
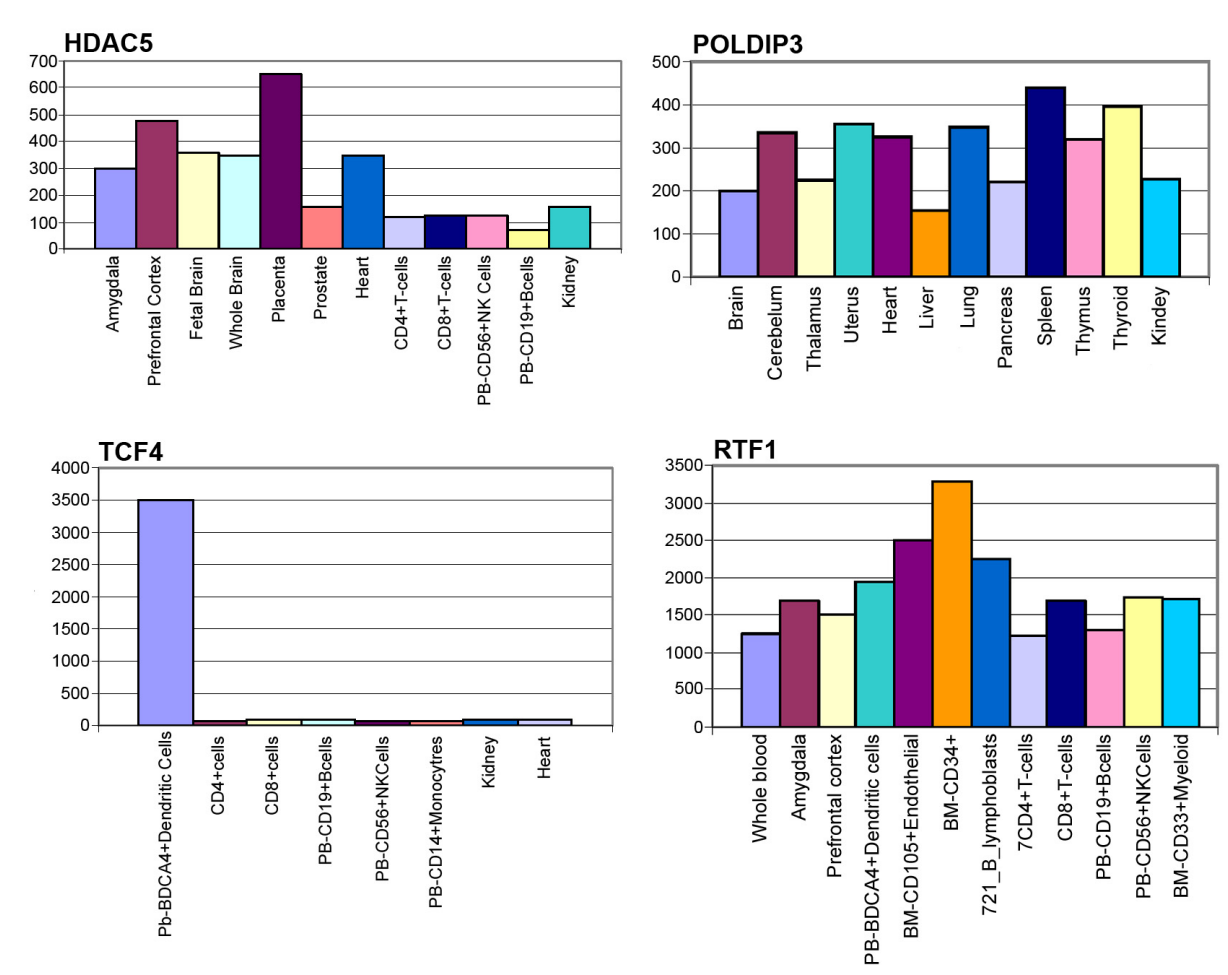
**Virtual northern blot of genes coding for autoantigens reported in this study.** Horizontal axis, tissues and cell lines; vertical axis, expression level of the given gene in arbitrary units of intensity.

The expression pattern of these genes in human was studied by a virtual Northern-blot analysis. Figure 1 shows the searching results. HDAC5 reaches a higher expression level in placenta, brain (both, adult and foetal), and heart. TCF4 is expressed at a medium level in most tissues with the exception of PB-BDCA4^+ ^(dendritic cell line) with a ten fold of the average expression level. RTF1 expression pattern shows the highest level in BM-CD34^+ ^cells and medium in endothelial tissue BMCD105 and PB-BDCA4^+ ^and T CD4^+ ^cell lines. POLDIP3 is the most evenly distributed protein among tissues, spleen is the predominant.

### The recombinant proteins

**Figure 2:**
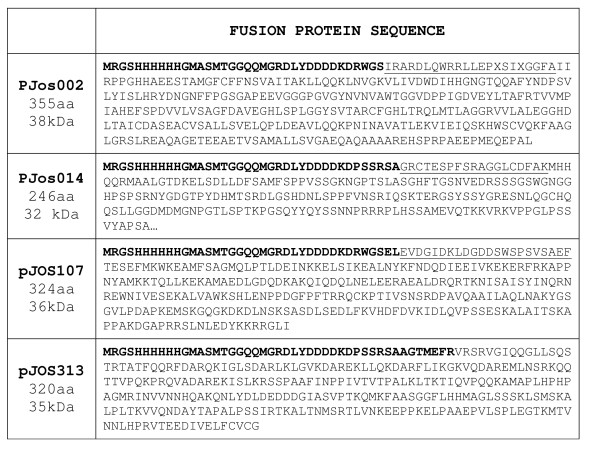
**Primary sequence of recombinant proteins used as antigens to check autoantibodies.** Amino acids in bold characters correspond to expressed part of cloning vector pRSET-B, underlined amino acids to λg11 phage.

Recombinant proteins sequences are shown in figure 2.

**Figure 3:**
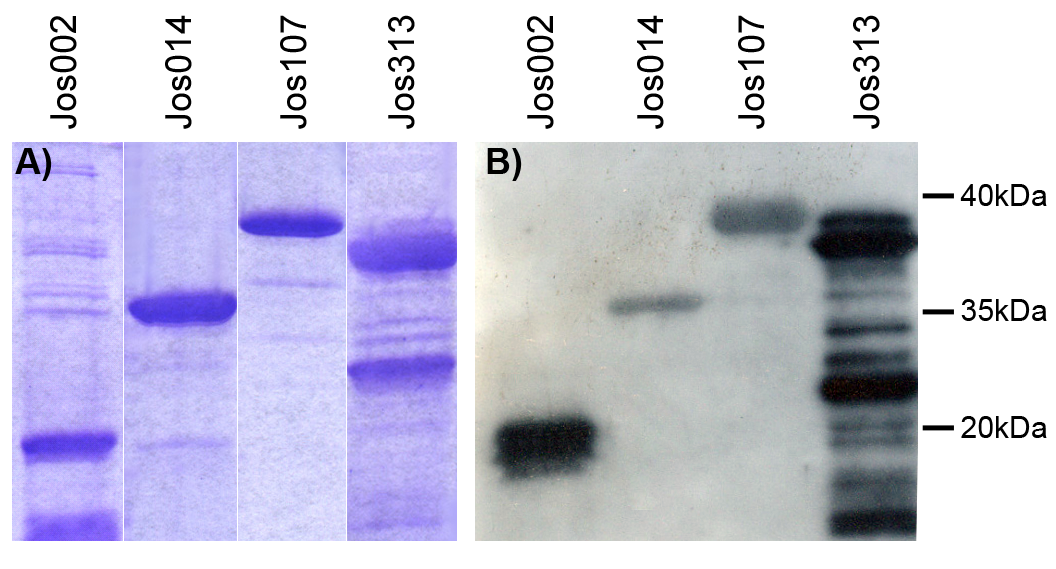
**(A) Recombinant proteins stained by coomassie blue.** Note the split peptide of Jos002 and multiple peptides in Jos313 (B) Western blot of recombinant proteins probed with serum from the patient origin of this study.

Recombinant protein Jos002 is formed by 355 residues with a mw of 38 kDa. Amino acids in bold characters (thirty-six) correspond to expressed part of cloning vector pRSET-B, underlined amino acids (twenty-two) to λg11 phage. Sequence Jos002 matches to HDAC5 C-terminus (from I_826 _to L_1122_). Figure 3 shows two smaller bands for this recombinant protein as result that *E. coli*, during the biosynthetic process splits this protein into two peptides of about 18 and 20 kDa.

Recombinant protein Jos014 is formed by 246 residues with a mw of 32 kDa. Amino acids in bold characters (thirty-eight) correspond to expressed part of cloning vector pRSET-A, underlined amino acids (nineteen) to λg11 phage. Sequence Jos014 matches to TFC4 N-terminus (from M_1 _to A_189_).

Recombinant protein Jos107 is formed by 324 residues with a mw of 36 kDa. Amino acids in bold characters (thirty-eight) correspond to expressed part of cloning vector pRSET-B, underlined amino acids (twenty-two) correspond to expressed part of λgt11 phage. Sequence Jos107 matches to Rtf1 (Paf1/RNA polymerase II complex component) C-terminus (T_167 _to I_429_).

Recombinant protein Jos313 is formed by 320 residues with a mw of 35 kDa. Amino acids in bold characters (forty-five) correspond to expressed part of cloning vector pRSET-A. Sequence Jos313 matches to Poldip3 (DNA polymerase delta interacting protein 3) (V_30 _to G_304_). Protein Jos313 is degraded by *E. coli *during the biosynthesis process into a non determined number of peptides, as shown in figure 3.

### Immunoreactivity of recombinant proteins the against patient's serum

Immunoreactivity of recombinant proteins described previously against patient's serum was checked by western-blot. Serum was diluted to 1:5000. Figure 3, panel B shows how these proteins are clearly recognized by the serum. As negative control in order to check serum cross-reactivity against usual *E. coli *products, the insert of clone Jos014 was expressed in the reverse orientation; as expected, an 8 kDa peptide was produced that was not recognized by any serum (data not shown).

### Prevalence and autoantibodies detection in sera from patients suffering from vasculitides

**Figure 4:**
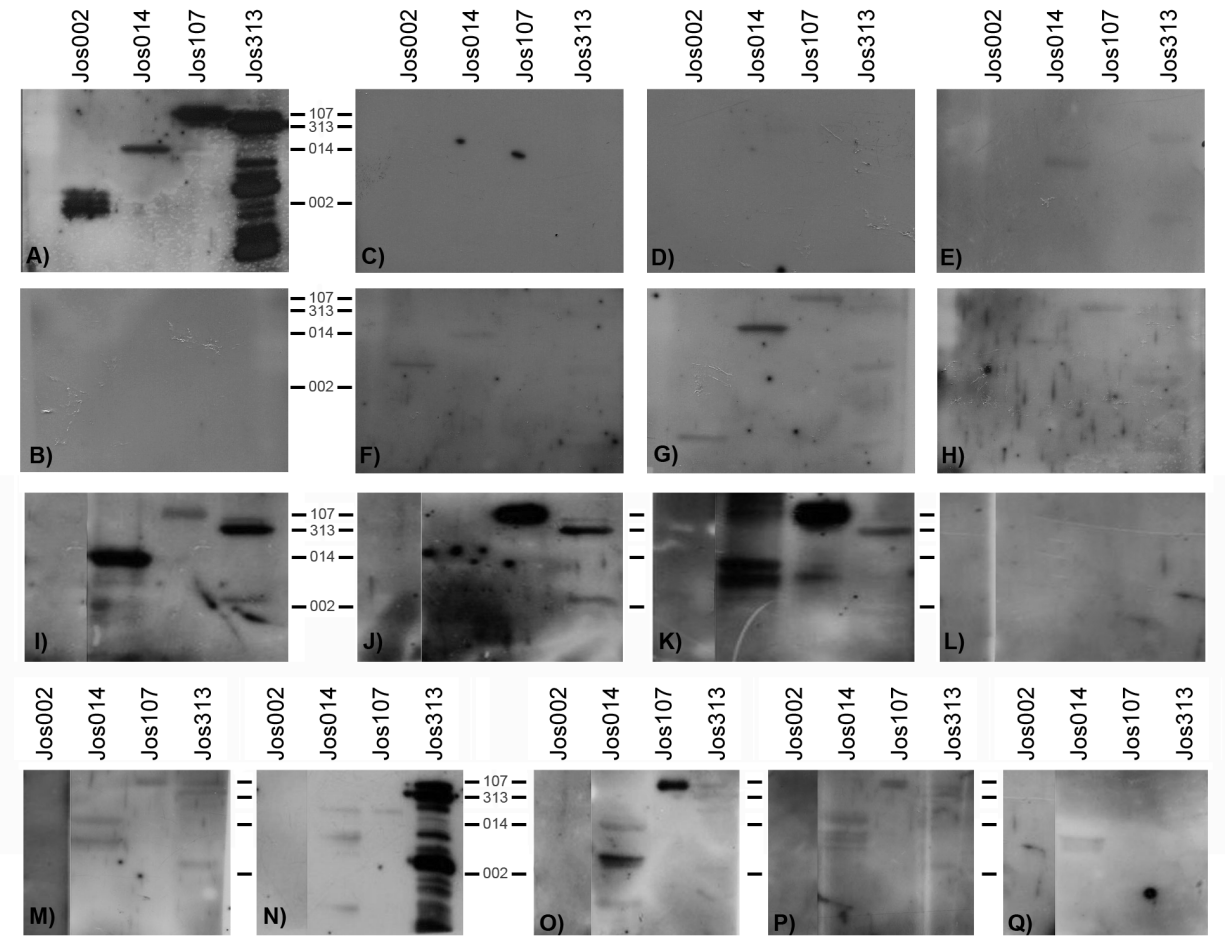
**Autoantibodies detection in controls and in sera from patients suffering of vasculitides. **Panel A shows a western blot probed with the serum from the patient subject of the study and panel B the western blot probed with serum from a representative healthy control. Panels C to H show western blot analysis of autoantibodies in several vasculitis patient's sera, suffering SLE (C to E), PAN (F to H and L), MPA (I), WG (J) and CSS (K). Panels M to Q show western blot analysis of autoantibodies of five patients suffering form diabetic microangiopathy. Specific molecular mass for each recombinant protein are shown between panels A and C, B and F, I and J and N and O.

Eighteen sera from apparently healthy people with no familial history of rheumatic diseases were tested by western blot for reactivity against all four recombinant proteins; none of them gave a positive signal, figure 4, panel B. This fact points out that none of them is a prevalent antigen. Sera from ten patients suffering of vasculitides (3 SLE, 4PAN, 1 WG, 1 CSS and 1 PMA) were also tested for reactivity by western blot against the recombinant proteins. Recombinant protein Jos002, a part of *HDAC5*, was recognized only for serum from one PAN patient (Figure 4, panel F), recombinant proteins Jos014, 107 and 313, were recognized in a scatter manner and with lower intensity than control (Josue's serum, Figure 4, panel A) by sera from some patients, as shown in figure 4, panels C to L.

### Prevalence of autoantibodies in renal glomerular diabetic microangiopathy

We checked the prevalence of autoantibodies against these proteins in patients suffering from a diabetic microangiopathy. None of these patients were positive for MPO or any ANCA. Results are shown in figure 4 panels M to Q. None of the sera reacted against proteins Jos002, two sera recognized Jos313 (Fig. 4, panels M and N), three out of five reacted against Jos107 and Jos014 (Fig. 4, panels M, O and P), sera from one patient did not react against any recombinant protein (Fig. 4, panel Q). These results might be indicative of cell destruction and autoantigens release and subsequent autoantibodies production. Whether the origin of antigens of broken cell nuclei is microvascular endothelial cells within a concurrent inflammatory process or any other affected cell remains to be elucidated.

Given the amount of cases studied there is no point of doing any statistical analysis, however, we can conclude that: a) none of the antibodies is prevalent in general population, b) autoantibodies against HDAC5 have low prevalence and c) autoantibodies against RTF1 and POLDIP3 are prevalent in both vasculitides and diabetic angiopathy.

### Autoantibodies and pathogenesis of nephro-glomerular vasculitis

We tried to reproduce some signs of this vasculitis, as glomerulonephritis or proteinury, by immunizing a series of mice (CB1/swiss species) with the recombinant proteins, either by injecting only one protein per mice or the four proteins together. Experimental procedures in mice were in accordance with the guidelines of the Animal Care Advisory Committee of the University of La Laguna. Sera from all animals were previously tested by western blots for possible auto antibodies prevalence against the antigens to test and proteins in urine; none of them gave a positive result. Six groups of four mice 5–6 weeks old each were made, four groups were immunized with 20 μg of the correspondent recombinant protein, the fifth with 10 μg of each protein, the sixth group, control, injected only with the vehicle and complete or incomplete Freund's adjuvant (complete day 0 and incomplete rest of immunizations). Mice were placed in metabolic cases and injected at days 0, 10, 20, 40, 56, 70 and 84. Urea, creatinine, Ca^2+^, Na^+^, K^+^, Cl^-^, phosphates and total proteins in both, serum and urine, were determined previous to injections. No significant changes from controls were found in any mice at any point of the study. Mice were sacrificed at day 90, sera were collected and kidneys saved for histological studies. Histological studies showed neither glomerular, nor tubular, nor interstitial nor minimal changes.

Western blots with sera from immunized mice showed reactivity against their correspondent recombinant proteins at titles as high as 1:5000. All this data together suggest that although the recombinant proteins elicited a good level of antibodies, these were not able to produce any damage, either morphological or functional, to the kidney.

To our knowledge, elegant and sophisticated experiments headed by Jennette and Falk [19,20] show that Rag2(-/-) mice injected intravenously with splenocytes from MPO immunized Mpo(-/-) knockout mice after immunisation with MPO protein has been shown to develop a glomerulonephritis, and systemic necrotizing vasculitis similar to humans.

## Conclusion

Autoantibodies against four proteins, HDAC5, TFC4, RTF1 and POLDIP3, observed in this study further confirms the autoimmune reactions in vasculitides and may provide new inroads into elucidating the immunopathogenesis of the disease. Most antibodies related to autoimmune diseases are considered as markers rather than etiopathogenic agents and, generally, are used as diagnostic tools associated to a determined disease based on statistics or epidemiological correlations. In the present study, additional assays to prove the involvement of the various autoantibodies in the pathogenesis and reproduce the disease in mice, however, did not support the hypothesis, either because the experimental model was developed in different species or because damaging antibodies were not generated or some epitopes were missing in the recombinant proteins. A long time study (years) reporting a larger amount of cases will give us the interest of autoantigens described in this article as predictive markers in vasculitides.

## Authors' contributions

JA performed library screening and made expression constructs, EA and MVM purified recombinant proteins and performed western blots, MFA performed western blots of first series controls, HC performed experiments with mice, EG and JJGP characterized and selected patients and controls and JA and PMV designed research, analyzed data and wrote the paper. All authors read and approved the final manuscript.

## List of abbreviations

ANA: Antinuclear antibody; ANCA: Neutrophile anticytoplasm antibody; c- or p- ANCA: Cytoplasmic or perinuclear-ANCA; BLAST: Basic local alignment search tool; bp: Base pairs; cDNA: DNA complementary to RNA; CSS: Churg-strauss syndrome; HDAC5: Histone deacetylase 5; IPTG: Isopropyl b-D-thiogalactopyranoside; kDa: Kilodaltons; PAN: Polyarteritis nodosa; MPA: Microscopic polyangiitis; MPO: Myeloperoxidase; PBS: Phosphate-buffered saline; PR3: Proteinase 3; POLDIP3: Polymerase -DNA-directed-delta interacting protein 3; RTF1: Gene for TATA site selection by TATA box-binding protein; SEREX: Serological identification of antigens by recombinant expression cloning; SLE: Systemic lupus erythematosus; TBS: Tris buffered saline; TFC4: Transcription factor 4; WG: Systemic Wegener's granulomatosis.

## Competing interests

The authors declare that they have no competing interests.
